# An Occlusion-Representation and Background-Redundancy-Suppression Network for Prohibited Item Detection with X-Ray Images

**DOI:** 10.3390/s26175569

**Published:** 2026-09-02

**Authors:** Hao Wen, Yanxi Zhang, Yanzu Huang, Hongji Xie, Xiangdong Gao

**Affiliations:** School of Electromechanical Engineering, Guangdong University of Technology, Guangzhou 510060, China; wenhao2@mails.gdut.edu.cn (H.W.); huangyazu@outlook.com (Y.H.); gran011019@gmail.com (H.X.); gaoxd@gdut.edu.cn (X.G.)

**Keywords:** prohibited item detection, X-ray security images, occlusion representation, background redundancy suppression, YOLO11-ORS

## Abstract

**Highlights:**

**What are the main findings?**

- YOLO11-ORS suppresses background redundancy and preserves occlusion-related structural cues in X-ray baggage images.
- YOLO11-ORS achieves 94.1% *mAP*@50 and 72.7% *mAP*@50:95 with 2.68 M parameters, 6.4 GFLOPs, and 156 FPS on SIXray.

**What are the implications of the main findings?**

- The proposed design improves robustness under object overlap, weak target visibility, and cluttered backgrounds.
- The model performs stably on both pseudo-color and grayscale X-ray images, supporting practical deployment.

**Abstract:**

Prohibited item detection in baggage X-ray images remains challenging because object overlap, partial occlusion, and cluttered backgrounds often obscure target boundaries. These factors make discriminative structural cues difficult to preserve in complex luggage scenes. This study proposes YOLO11-ORS, an occlusion representation and background redundancy suppression network built upon YOLO11n. The proposed model improves feature representation in two ways. First, a Background Redundancy Suppression Pyramid (BRSP) module is designed after semantic feature aggregation to refine contextual features at multiple scales and suppress irrelevant responses from cluttered baggage contents. Second, an Occluded Structure Enhancement Module (OSEM) is constructed at the attention-based feature transformation stage to preserve weak edge cues and incomplete local structures of partially occluded prohibited items. Experiments on the SIXray dataset show that YOLO11-ORS achieves 94.1% *mAP*@50 and 72.7% *mAP*@50:95, improving the YOLO11n baseline by 1.2 and 0.9 percentage points. On the OPIXray dataset, YOLO11-ORS improves *mAP*@50 and *mAP*@50:95 over YOLO11n by 1.2 and 0.8 percentage points, respectively, while the *mAP*@50:95 gain reaches 1.5 percentage points under severe occlusion (OL3). Evaluations on difficult test subsets further demonstrate its robustness under cluttered backgrounds, object overlap, and weak target visibility. Additional validation on a self-collected grayscale X-ray dataset confirms its stable performance under different imaging conditions. Overall, YOLO11-ORS improves detection reliability in complex X-ray security inspection scenarios while maintaining practical inference efficiency.

## 1. Introduction

The rapid growth of transportation and logistics has increased the demand for efficient security screening in public transport hubs [1,2]. As a nondestructive imaging technique that can reveal the contents inside parcels, X-ray imaging has become a key means for prohibited item inspection [3]. However, routine screening still relies heavily on manual interpretation of pseudo-color X-ray images by security personnel. In high-throughput scenarios involving large numbers of passengers and pieces of luggage, inspectors often work under considerable pressure. Under such conditions, visual fatigue, distraction, and differences in personal experience can easily lead to missed detections and false positives, thereby posing risks to public safety [4,5]. Therefore, developing automated prohibited item detection systems that combine high accuracy, rapid processing, and real-time capability has become a key concern in the fields of computer vision and public security.

In recent years, object detection has been substantially promoted by deep learning, particularly by approaches developed from Convolutional Neural Networks (CNNs) [6,7]. Current detection methods are commonly classified into two categories. One category consists of two-stage detectors, with Faster Region-based Convolutional Neural Network (Faster R-CNN) as a typical representative [8]. The second group is one-stage detectors, represented by the Single Shot MultiBox Detector (SSD) [9] and the You Only Look Once (YOLO) family [10]. Although two-stage detectors usually deliver competitive accuracy, the region proposal process introduces considerable computational overhead and limits inference speed, which reduces their practicality in real-time security inspection scenarios [11]. In contrast, YOLO-based models complete detection in an end-to-end manner and therefore provide a more suitable balance between accuracy and efficiency, making them widely used in industrial applications [12]. A systematic review further indicates that lightweight YOLO variants are particularly promising for deployment on edge devices with constrained computing resources [13].

Although deep learning has achieved strong performance in general visual tasks, its direct use in X-ray security inspection still faces several notable challenges [14]. A primary difficulty is severe occlusion and object overlap. Since X-ray imaging is formed through penetrative projection, objects with different material properties and densities inside a package are projected onto the same 2D image plane, which often blurs the boundaries of prohibited items and causes their texture information to be obscured by surrounding clutter [15,16]. To mitigate this issue, Miao et al. constructed the SIXray dataset and proposed a Class-balanced Hierarchical Refinement (CHR) graph neural network for overlapped object detection [17], whereas Wei et al. released the OPIXray dataset and introduced an edge-guided mechanism to separate mutually occluding objects [18]. Another important challenge is scale variation together with the low detectability of small targets. In security inspection imagery, prohibited items may differ markedly in size, and some targets occupy only a very limited number of pixels [19]. In response, Liang et al. showed that combining multi-scale feature fusion with spatial context extraction can effectively alleviate the degradation caused by small-object characteristics and occlusion interference [20]. Wang et al. [21] developed a multi-scale feature fusion scheme that combines deep semantic representations with low-level fine-detail information, thereby improving the identification of small prohibited targets in X-ray images. Fang et al. [22] also introduced a reweighted loss function for small and overlapping samples, encouraging the detector to focus more on difficult instances. Moreover, studies conducted in practical security screening environments have shown that adapting CNN architectures to the imaging characteristics of X-ray data is crucial for enhancing feature extraction in complex background conditions [23,24].

To improve prohibited item detection under object overlap and redundant background interference, this work develops an improved detection network based on YOLOv11n, termed YOLO11-ORS. The proposed model focuses on two task-driven feature optimization strategies: suppressing redundant background responses in cluttered X-ray images and preserving incomplete structural cues of occluded prohibited items. The main contributions of this paper are as follows:

(1) An occlusion representation and background redundancy suppression network, named YOLO11-ORS, is proposed for prohibited item detection in X-ray security images. The model aims to improve detection robustness under object overlap, weak target visibility, and complex baggage backgrounds.
(2) The BRSP module is constructed to reduce background interference. It enhances the discrimination of deep semantic features and suppresses redundant responses caused by cluttered luggage contents.
(3) The OSEM is designed to alleviate feature loss caused by object overlap and partial occlusion. It strengthens the representation of weak edge cues and incomplete structural information of prohibited items.
(4) Extensive experiments on SIXray and OPIXray, together with the selected SIXray difficult test subsets and auxiliary validation on the self-collected XPI8 Dataset, verify the effectiveness and robustness of YOLO11-ORS under general and challenging inspection scenarios.

## 2. Proposed Method

### 2.1. YOLO11-ORS Model

YOLOv11 is an efficient one-stage detector that follows the typical backbone–neck–head architecture. It improves feature extraction and feature fusion across scales while maintaining a relatively low computational cost [25]. Among the YOLO11 variants, YOLO11n has the smallest model size and the lowest computational burden. Therefore, it is selected as the baseline model in this study to meet the requirements of real-time X-ray security inspection on devices with limited computing resources. The comparative experiments in Section 4.3 further examine this choice against other lightweight YOLO variants. YOLO11n achieves competitive overall detection accuracy and the highest Recall among the evaluated YOLO baselines while maintaining a compact parameter scale and computational cost, providing a suitable foundation for investigating the proposed task-oriented feature optimization modules.

However, X-ray prohibited item detection is still affected by object overlap, partial occlusion, weak target boundaries, and complex baggage backgrounds. To address these problems, this study develops YOLO11-ORS based on YOLO11n. The proposed network modifies the backbone from two task-oriented perspectives: semantic recalibration for background redundancy suppression and occlusion-oriented feature transformation for structural cue preservation.

**Figure 1:**
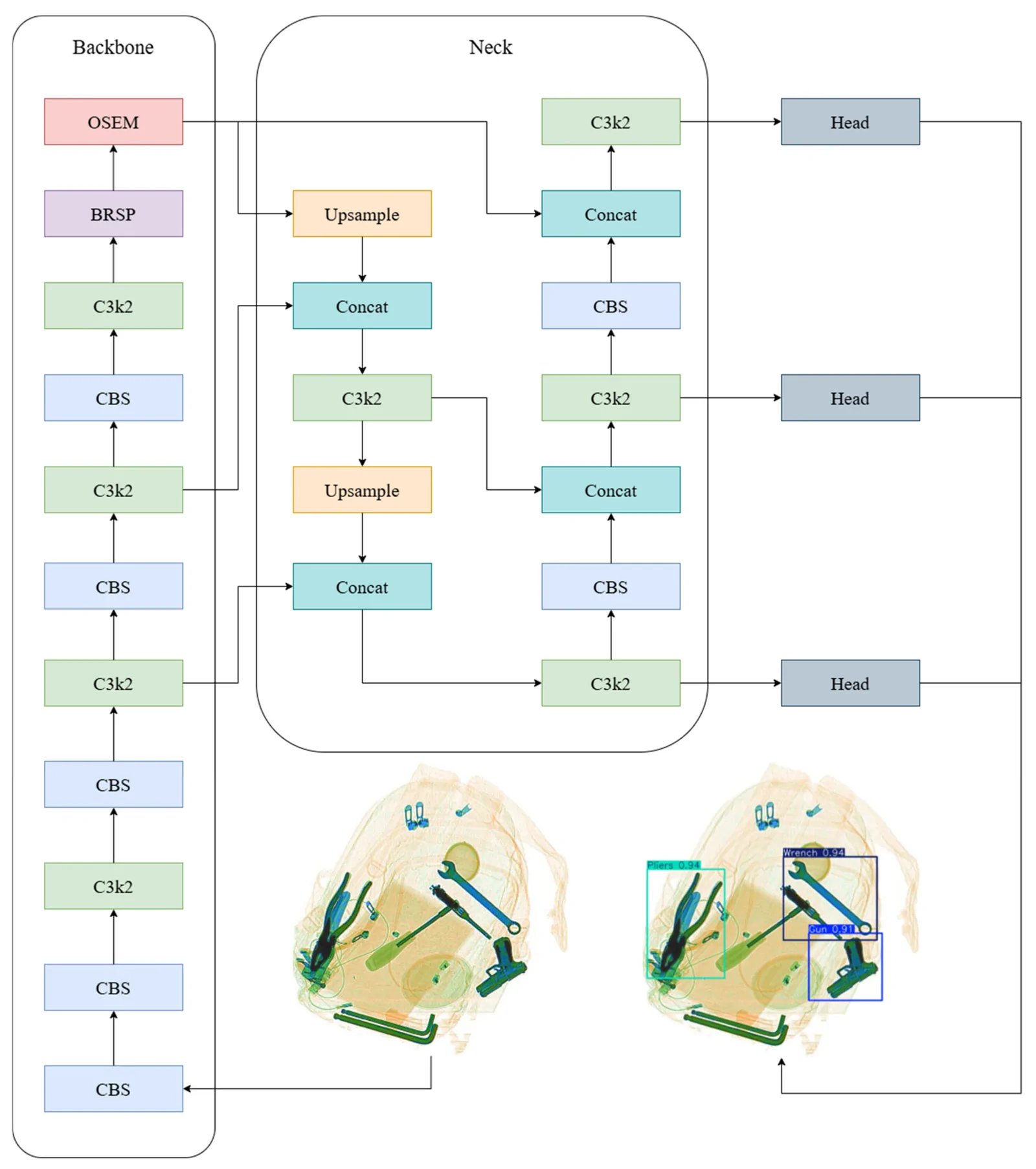
Network structure of YOLO11-ORS.

Specifically, BRSP is designed after semantic feature aggregation to enhance feature discrimination and suppress redundant background responses caused by cluttered luggage contents. OSEM is constructed at the attention-based feature transformation stage to strengthen weak edge cues and incomplete structural information of partially occluded prohibited items. Through these coordinated designs, YOLO11-ORS improves feature representation for complex X-ray security images while retaining the lightweight and real-time characteristics of YOLO11n. The overall architecture of YOLO11-ORS is shown in Figure 1.

### 2.2. BRSP Module

In X-ray security images, prohibited items are often superimposed with dense baggage contents, object stacking, and material overlap, which may introduce redundant background responses into deep semantic features. Although multi-scale feature aggregation provides richer contextual information, it may also preserve weak or less discriminative activations originating from surrounding non-target structures. These background-related responses can reduce the separability between prohibited items and cluttered baggage contents. To alleviate this problem, we construct a Background Redundancy Suppression Pyramid (BRSP) at the rear stage of the YOLO11n backbone.

BRSP extends the conventional Spatial Pyramid Pooling Fast (SPPF) structure by introducing a Grouped Convolutional Block Attention Enhancement (GCE) mechanism [26] after multi-scale feature aggregation. Rather than proposing a new generic attention operator, BRSP combines the contextual aggregation capability of SPPF with grouped channel–spatial recalibration to refine deep semantic features specifically for cluttered X-ray scenes.

**Figure 2:**
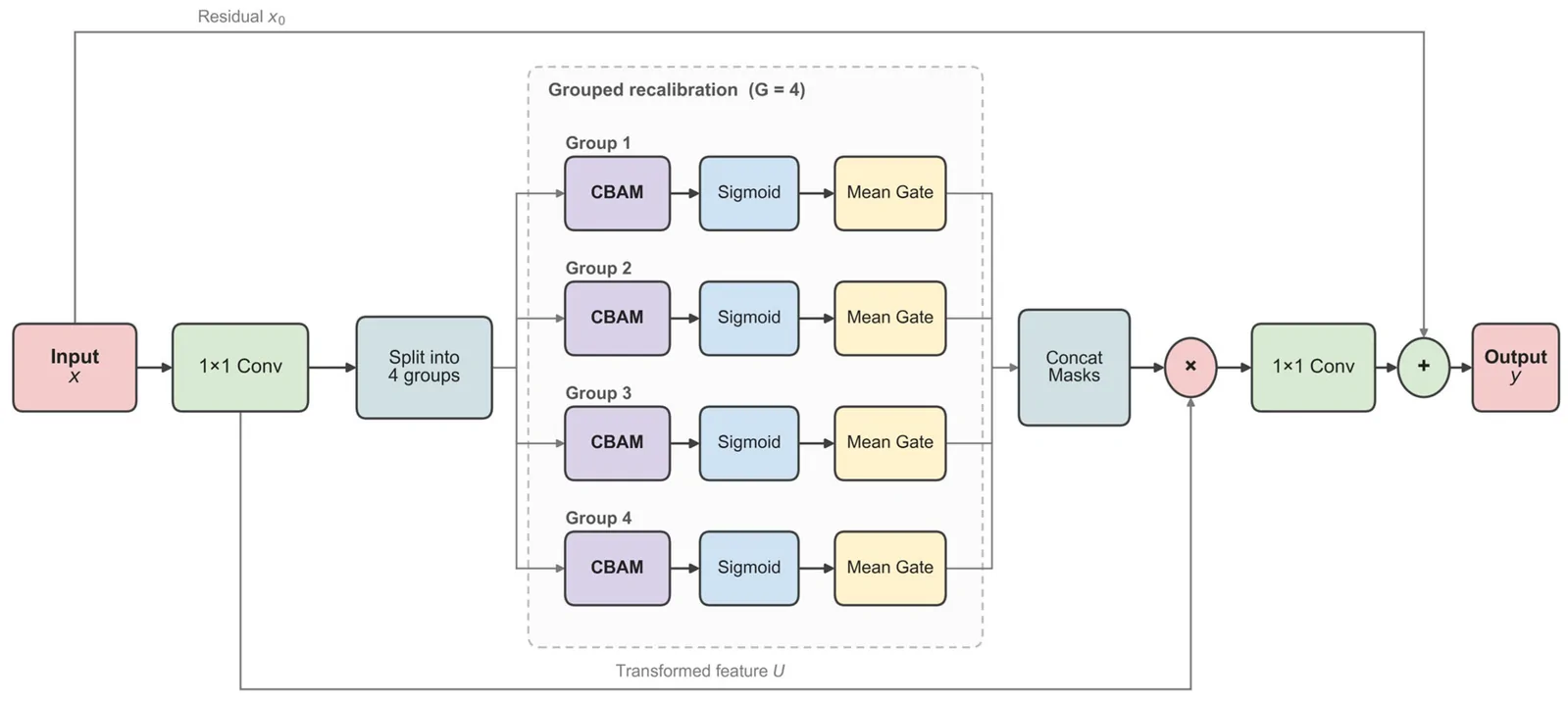
Architecture of the GCE module.

As illustrated in Figure 2, GCE first applies a 1×1 convolution to obtain a transformed feature U, which is divided into four channel groups. Each group is independently processed by a lightweight CBAM branch and then mapped through a Sigmoid function. An intra-group mean-gating strategy is subsequently applied:

(1) $$M_{g}(i)=\left\{\begin{array}{c} 1,\;\;\;\;\;\;S_{g}(i)>\mu_{g}\\ S_{g}(i),S_{g}(i)\leq \mu_{g} \end{array}\right.\;\mu_{g}=Mean(S_{g})$$

where $S_{g}(i)$ denotes the response of the i-th element in the -th feature group, and $\mu_{g}$ is the corresponding group-wise mean. Responses above the group mean are preserved without attenuation, whereas relatively low responses are adaptively weakened according to their Sigmoid values. Therefore, the mean gate acts as a selective preservation-and-attenuation mechanism rather than directly amplifying high-response features. The gating masks from all groups are then concatenated and multiplied element-wise with the transformed feature U. A subsequent 1×1 convolution performs cross-group feature fusion, followed by a residual connection with the original GCE input X, which helps avoid excessive information loss during recalibration.

**Figure 3:**
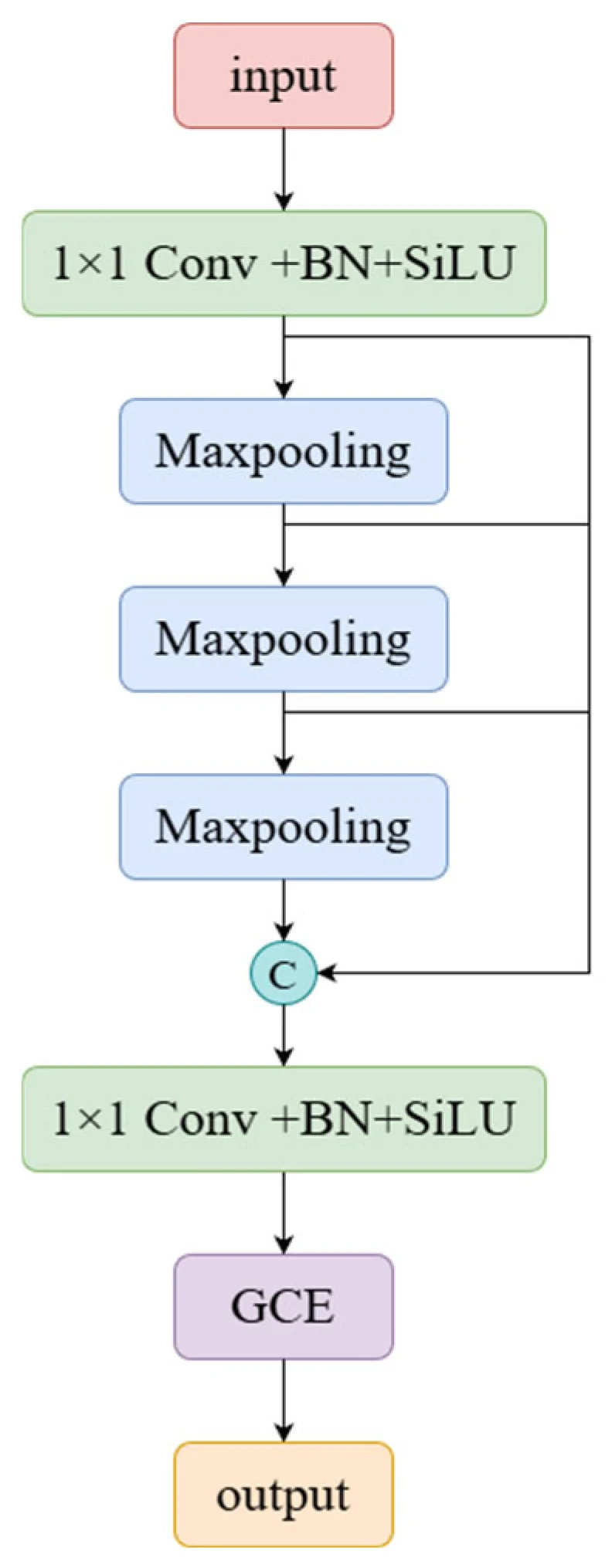
Schematic of the BRSP module.

As shown in Figure 3, BRSP retains the multi-scale aggregation mechanism of SPPF. The input feature is first compressed by a 1×1 Conv-BN-SiLU block, followed by three cascaded 5×5 max-pooling operations with stride 1. The resulting multi-scale features are concatenated and projected to the output channel dimension, after which GCE is applied for further semantic refinement.

Through this design, BRSP extends SPPF from pure multi-scale spatial aggregation to background-oriented semantic recalibration. The SPPF branch provides broader contextual information, while GCE redistributes channel–spatial responses through grouped attention and intra-group gating. In cluttered X-ray baggage images, object stacking, material overlap, and non-target contents may introduce weak or less discriminative responses into deep feature maps. The proposed gating strategy is intended to reduce the contribution of relatively weak responses associated with cluttered background interference while preserving more salient semantic structures. As a result, target-related features become more distinguishable from surrounding baggage contents, thereby improving deep feature discrimination under complex X-ray backgrounds.

### 2.3. OSEM

Partial occlusion is another major challenge in X-ray prohibited-item detection. Due to the penetrative projection mechanism of X-ray imaging, prohibited items are frequently overlapped by surrounding baggage contents. Under moderate or severe occlusion, only fragmented edges, partial contours, or incomplete structural patterns may remain visible. These weak but discriminative cues can be progressively diluted during deep feature transformation, making partially visible targets more difficult to localize and classify.

To improve the representation of such incomplete structural information, we construct an Occluded Structure Enhancement Module (OSEM) by integrating the existing Separation and Enhancement Attention Module (SEAM) [27] into the Cross Stage Partial with Spatial Attention (C2PSA) structure. The contribution of OSEM does not lie in introducing SEAM itself; instead, SEAM is embedded into the feature-transformation stage of PSABlock so that local structural modeling and channel-selective enhancement can be introduced for occlusion-oriented feature representation.

**Figure 4:**
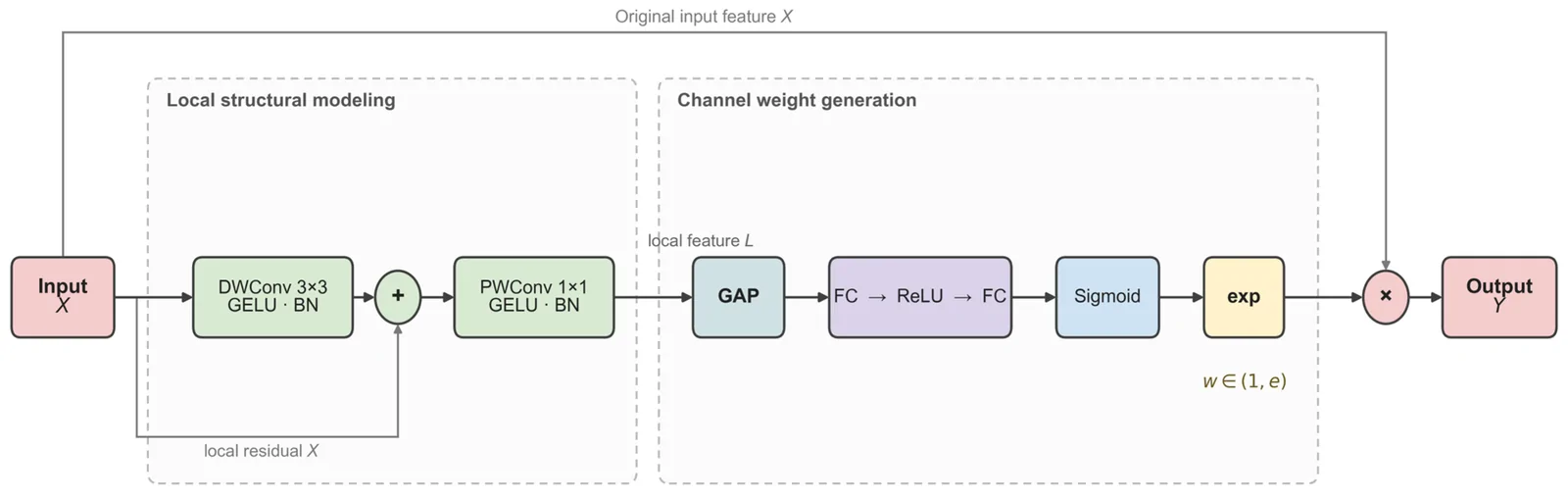
Architecture of the SEAM feature transformation used in OSEM.

As illustrated in Figure 4, SEAM first performs local structural modeling using a residual 3×3 depthwise convolution, followed by GELU activation and Batch Normalization. A subsequent 1×1 pointwise convolution is used for cross-channel interaction, again followed by GELU and Batch Normalization. The depthwise operation models local neighborhood information with low computational overhead, while the pointwise convolution integrates the locally extracted responses across channels. The resulting locally transformed feature is denoted as L.

The locally transformed feature is then compressed by global average pooling and passed through a two-layer fully connected network to generate channel weights. SEAM further applies exponential mapping after Sigmoid normalization, and the main transformation can be summarized as:

(2) $$Y=X⊙exp\left[\sigma (W_{2}\delta (W_{1}GAP(L)))\right]$$

where Y denotes the output feature, $W_{1}$ and $W_{2}$ represent the two fully connected transformations, δ and σ denote ReLU and Sigmoid, respectively, and ⊙ represents element-wise multiplication.

Since the Sigmoid output lies in $\left(0,1\right)$, exponential mapping expands the resulting channel-weight range to $\left(1,e\right)$. Therefore, SEAM performs channel-selective amplification rather than explicit spatial suppression. The channel weights are generated from the locally transformed feature L but are finally multiplied with the original input feature X. Consequently, channels assigned larger scores based on the locally modeled representation receive stronger amplification, while the original feature information is retained.

**Figure 5:**
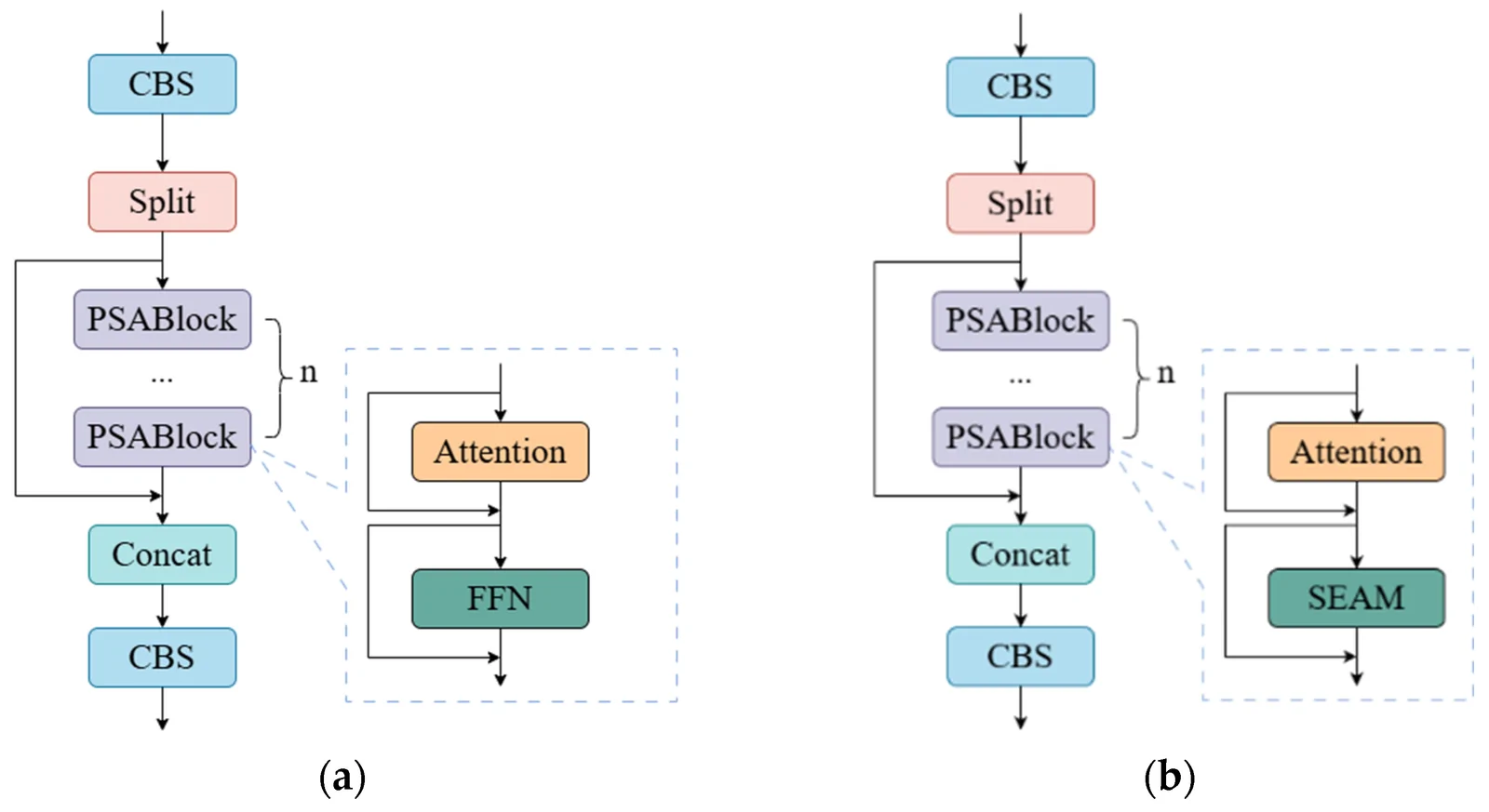
Comparison between the original C2PSA and the proposed OSEM. (**a**) Original C2PSA; (**b**) OSEM, in which the feed-forward network of PSABlock is replaced by SEAM while the original attention pathway is retained.

As illustrated in Figure 5, OSEM retains the original attention pathway of PSABlock, while replacing its feed-forward network with SEAM. Multiple SEAM-enhanced PSABlocks are then organized within the C2PSA framework to form the modified feature-transformation stage. In this way, local structural modeling and channel-selective enhancement are introduced without removing the original spatial-attention operation.

Through this design, OSEM introduces occlusion-oriented structural enhancement into the deep attention-based representation stage. The depthwise–pointwise branch models local patterns such as fragmented edges and partial contours, while the subsequent exponential weighting preferentially amplifies channels assigned larger scores based on the locally transformed representation. This mechanism is intended to preserve informative structural cues that may otherwise be weakened by target overlap and occlusion, thereby improving the representation of partially visible prohibited items during deep feature transformation.

## 3. Experimental Setup

### 3.1. Datasets

Three X-ray image datasets were used for model evaluation: the public SIXray dataset, the public OPIXray dataset, and the X-ray Prohibited-item Dataset with 8 categories (XPI8 Dataset). In addition, three difficult test subsets were constructed from the SIXray test set for robustness evaluation.

#### 3.1.1. SIXray Dataset

**Figure 6:**
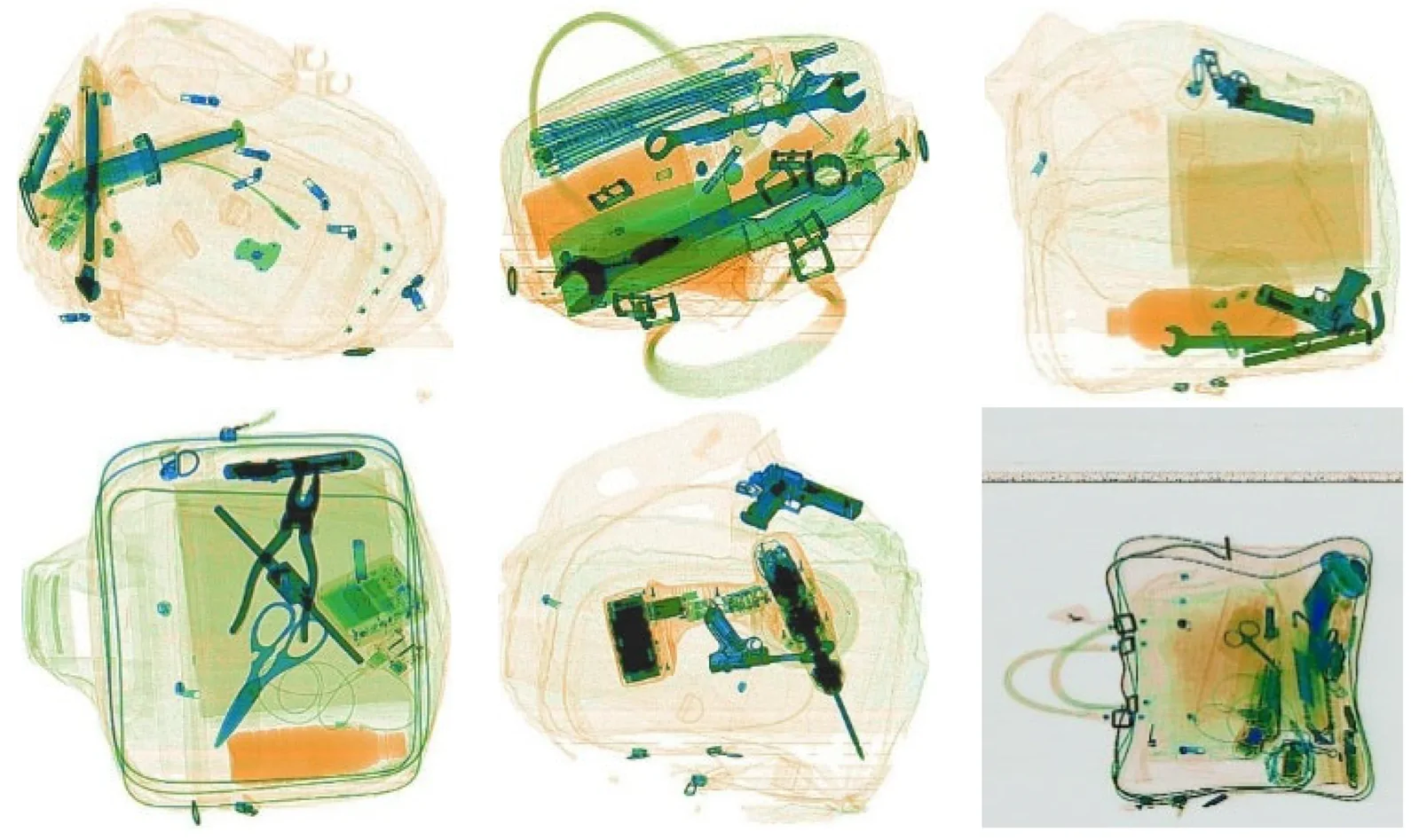
Sample images from SIXray.

SIXray is currently among the most widely adopted large-scale public datasets used in X-ray security screening. It is collected from real luggage and parcels passing through security screening channels in the Beijing Subway system via a multi-view X-ray imaging system. The images are rendered using dual-energy pseudo-color imaging technology. The image samples in the SIXray dataset effectively reflect material differences among organic substances, inorganic substances, and metals, while also encompassing abundant information on object stacking, occlusion, and complex backgrounds [17]. Figure 6 presents several example samples from SIXray.

**Figure 7:**
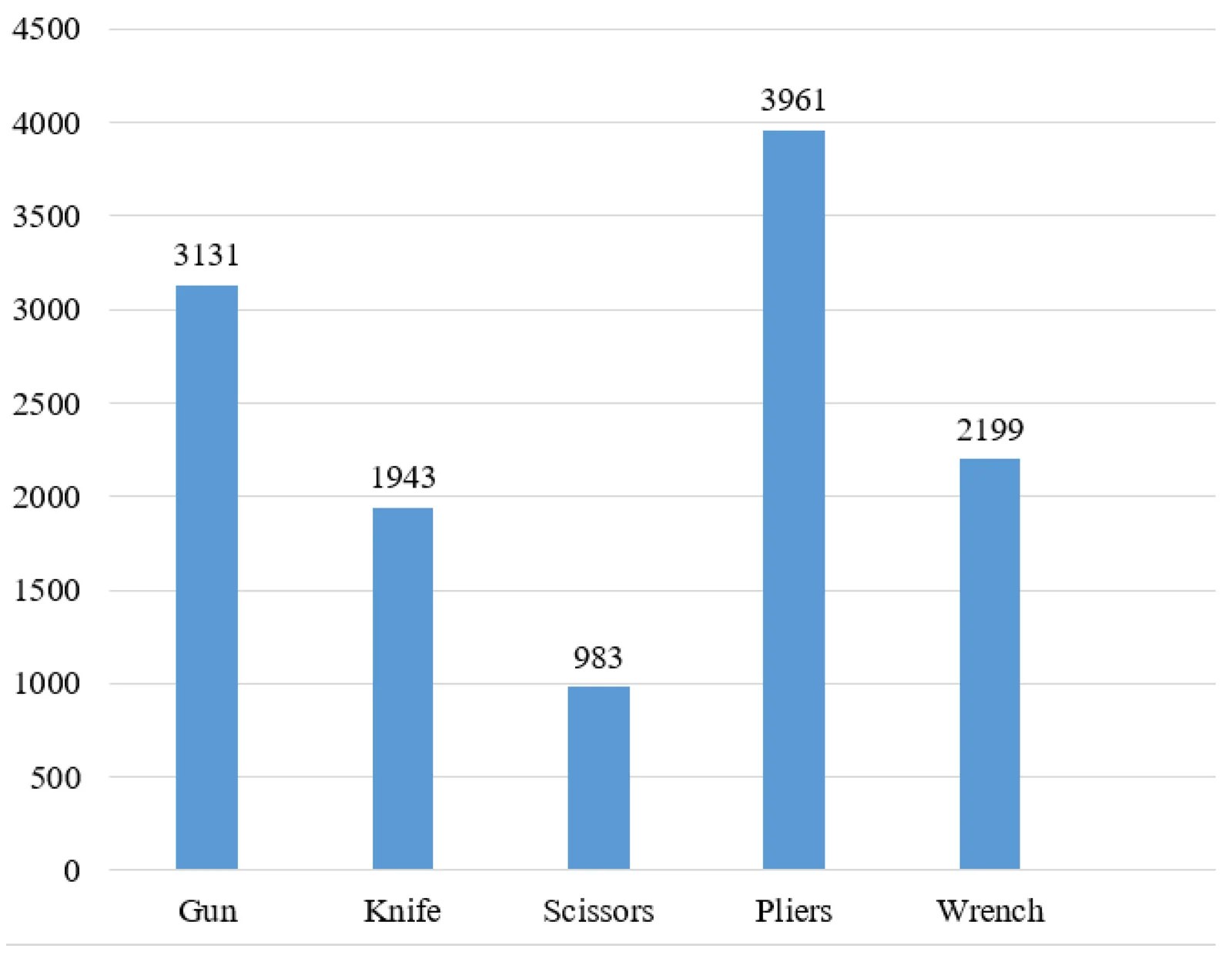
Category-wise instance distribution in the SIXray dataset.

The dataset contains 1,059,231 X-ray images, of which 8929 are annotated as prohibited items. The positive samples are distributed across six categories, including Gun, Knife, Wrench, Pliers, Scissors, and Hammer. The remaining 1,050,302 images are unlabeled negative samples. Since this study focuses on supervised object detection with bounding-box annotations, only annotated positive images were used for training, validation, and testing. In addition, categories with extremely limited samples may affect training stability. Therefore, the Hammer class is excluded from the experiments. Accordingly, five prohibited item categories are retained for the final experiments. The data are divided into training, validation, and test subsets using a 7:2:1 ratio for model development, validation, and final evaluation. The category-wise instance distribution is shown in Figure 7.

#### 3.1.2. Difficult Test Subsets

To evaluate the robustness of the proposed model under challenging X-ray inspection conditions, three difficult subsets were extracted from the original SIXray test set, namely the small-object subset, the overlap subset, and the cluttered-background subset. These samples were selected only from the test split and were not used for training or validation.

The small-object subset was selected according to the minimum target area ratio, defined as the ratio between the smallest bounding-box area and the whole image area. Images whose minimum target area ratio fell within the lowest 30% of all target-box area ratios in the test set were retained. The overlap subset was constructed using the maximum pairwise overlap ratio between annotated boxes, calculated as the intersection area divided by the smaller bounding-box area. Images with a maximum overlap ratio no lower than 0.20 were selected to represent object overlap and partial occlusion.

For the cluttered-background subset, target regions were first masked to avoid counting target edges as background information. Background complexity was then measured using edge density, Laplacian variance, and connected-component density. After robust normalization using the 5th and 95th percentiles, the clutter score was calculated using Equation (3):

(3) $$S_{clutter}=0.45S_{edge}+0.35S_{lap}+0.20S_{comp}$$

where $S_{edge}$, $S_{lap}$, and $S_{comp}$ denote the normalized edge density, Laplacian variance, and connected-component density, respectively. Images with clutter scores in the top 30% of the test set were selected as cluttered-background samples.

The coefficients in Equation (1) are fixed empirical weights rather than trainable parameters. Edge density was assigned the largest weight because it directly reflects the abundance of contour structures in cluttered baggage backgrounds. Laplacian variance was used as a complementary measure of local intensity and texture variation, but it is relatively more sensitive to imaging noise and contrast. Connected-component density characterizes the fragmentation of background structures; however, it is affected by the edge-detection threshold and partially overlaps with the information represented by edge density. Therefore, weights of 0.45, 0.35, and 0.20 were assigned to the normalized edge density, Laplacian variance, and connected-component density, respectively. The coefficients sum to one, and they were predefined without using detector predictions or detection performance on the test subset.

For each subset, candidate images were ranked by the corresponding difficulty score, and at most 300 images were retained. The overall difficult subset was obtained by merging the three selected subsets and removing duplicate images. Therefore, the constructed difficult subsets correspond to three typical challenges in X-ray prohibited item detection: weak target visibility, object overlap, and redundant background interference.

**Figure 8:**
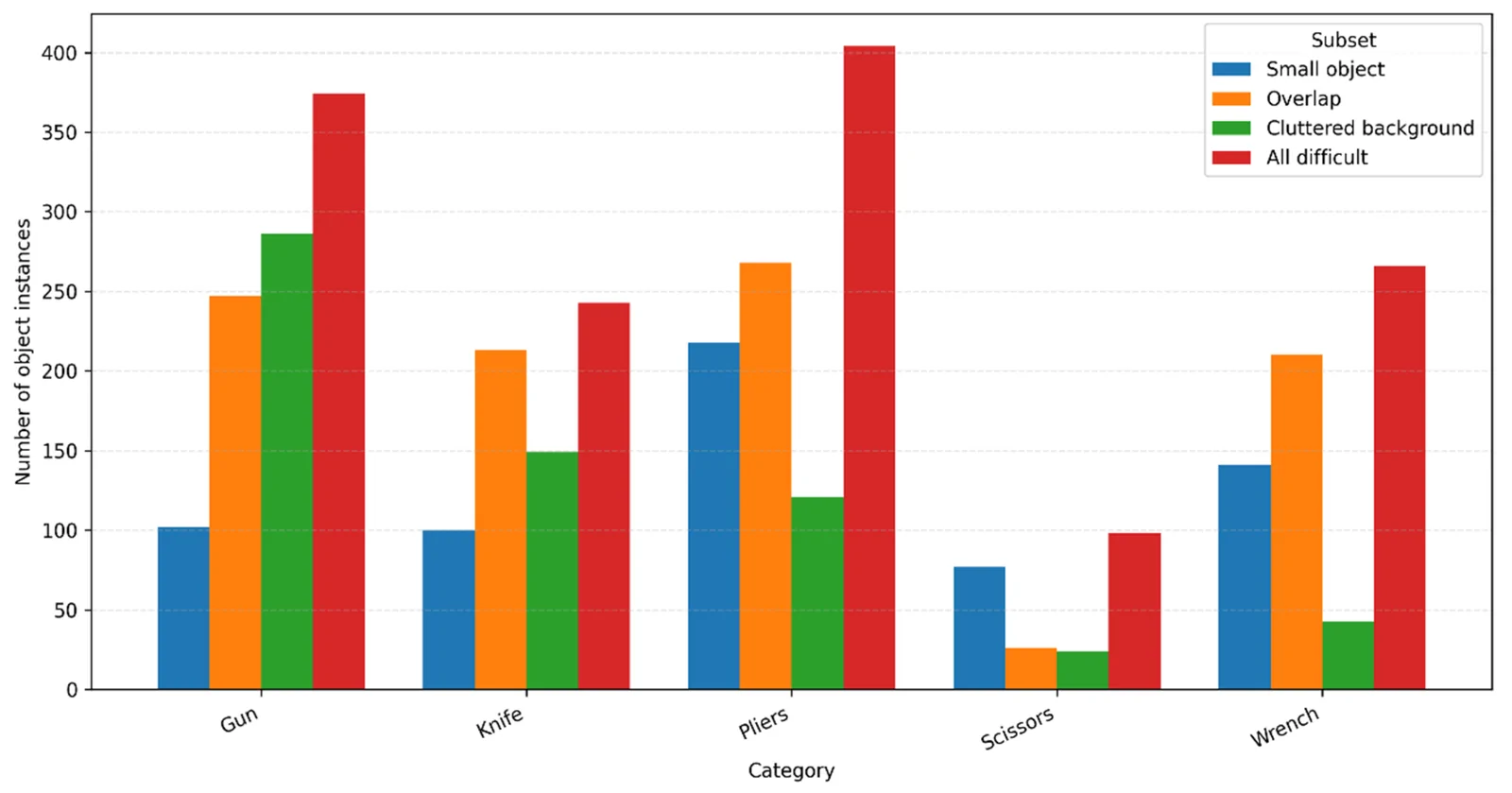
Category distribution of selected difficult test subsets.

Figure 8 shows the category distribution of the selected difficult test subsets. The statistics refer to object instances rather than image numbers. The small-object, overlap, and cluttered-background subsets contain 638, 964, and 623 object instances, respectively. After duplicate images were removed, the overall difficult subset contained 1385 object instances across all five prohibited item categories, indicating that the selected difficult samples are not concentrated in a single class.

**Figure 9:**
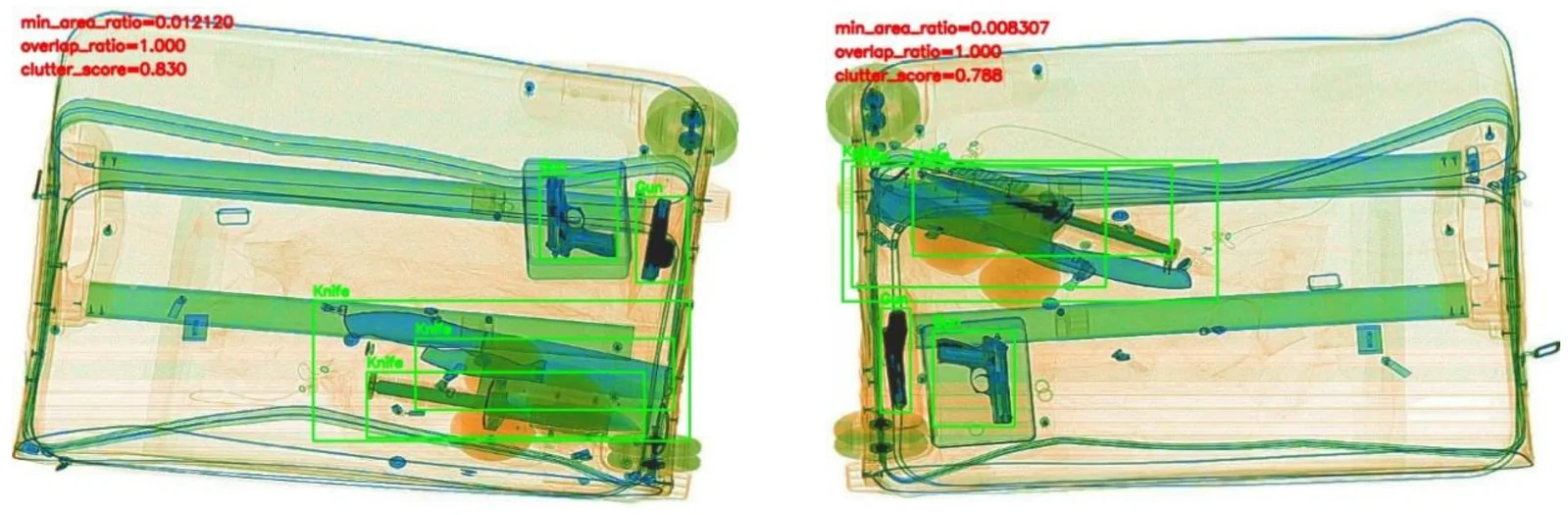
Visualization examples from selected difficult samples.

Figure 9 presents visualization examples from the selected difficult samples. The green bounding boxes and category names denote the ground-truth annotations, while the red values indicate the calculated difficulty indicators, including min_area_ratio, overlap_ratio, and clutter_score.

#### 3.1.3. OPIXray Dataset

OPIXray is a public X-ray security-inspection dataset designed for occluded prohibited-item detection [18]. It contains 8885 X-ray images from five categories of prohibited cutters: Folding Knife, Straight Knife, Scissor, Utility Knife, and Multi-tool Knife. The official dataset provides predefined training and test sets. In this study, part of the original training set was further separated to construct an independent validation subset, resulting in an approximate train/validation/test ratio of 7:1:2, while the official test set was kept unchanged for final evaluation.

**Figure 10:**
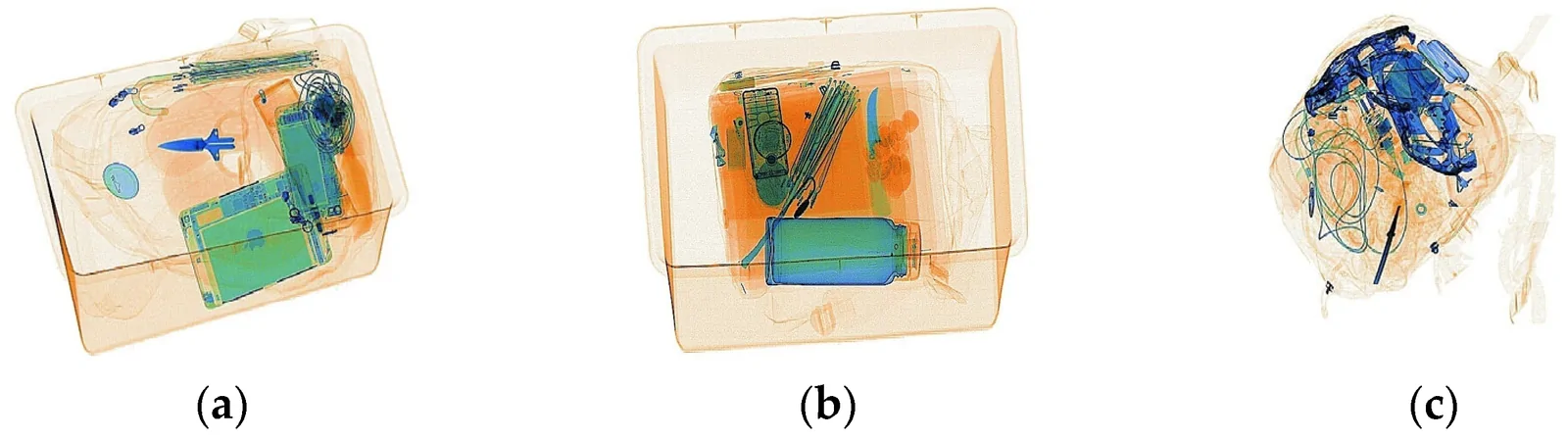
Representative samples from the OPIXray dataset under different occlusion levels: (**a**) OL1, no or slight occlusion; (**b**) OL2, partial occlusion; and (**c**) OL3, severe or nearly complete occlusion.

A distinctive feature of OPIXray is that its test set is divided into three subsets according to the degree of target occlusion: Occlusion Level 1 (OL1), Occlusion Level 2 (OL2), and Occlusion Level 3 (OL3), corresponding to increasing occlusion severity. Therefore, in addition to evaluation on the complete test set, the models were separately tested on OL1–OL3 to investigate their robustness under different occlusion conditions. Representative samples from OPIXray are shown in Figure 10.

#### 3.1.4. XPI8 Dataset

**Figure 11:**
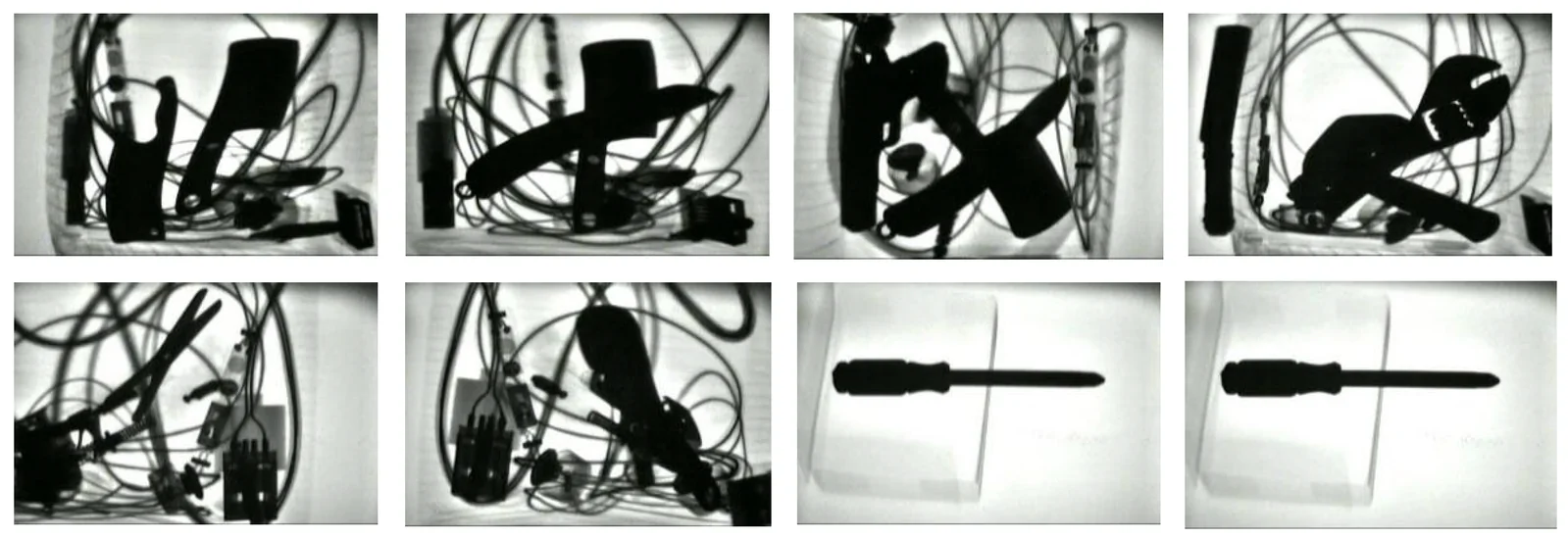
Example images from XPI8 Dataset.

A self-collected grayscale X-ray dataset, denoted as XPI8 Dataset, was established to further evaluate the model under different imaging settings. The dataset was acquired using an X-ray scanner operating at a relatively low radiation dose, and transmission imaging was performed on common objects found in security inspection scenarios. Unlike SIXray, which consists of dual-energy pseudo-color images, the XPI8 Dataset is composed of single-energy grayscale images, resulting in a clear domain difference between the two datasets. This dataset is designed to reflect practical security-inspection situations, including cases with target overlap and occlusion. Figure 11 presents some examples from the XPI8 Dataset. Compared with SIXray, however, the XPI8 Dataset has simpler backgrounds and lower overall scene complexity.

**Figure 12:**
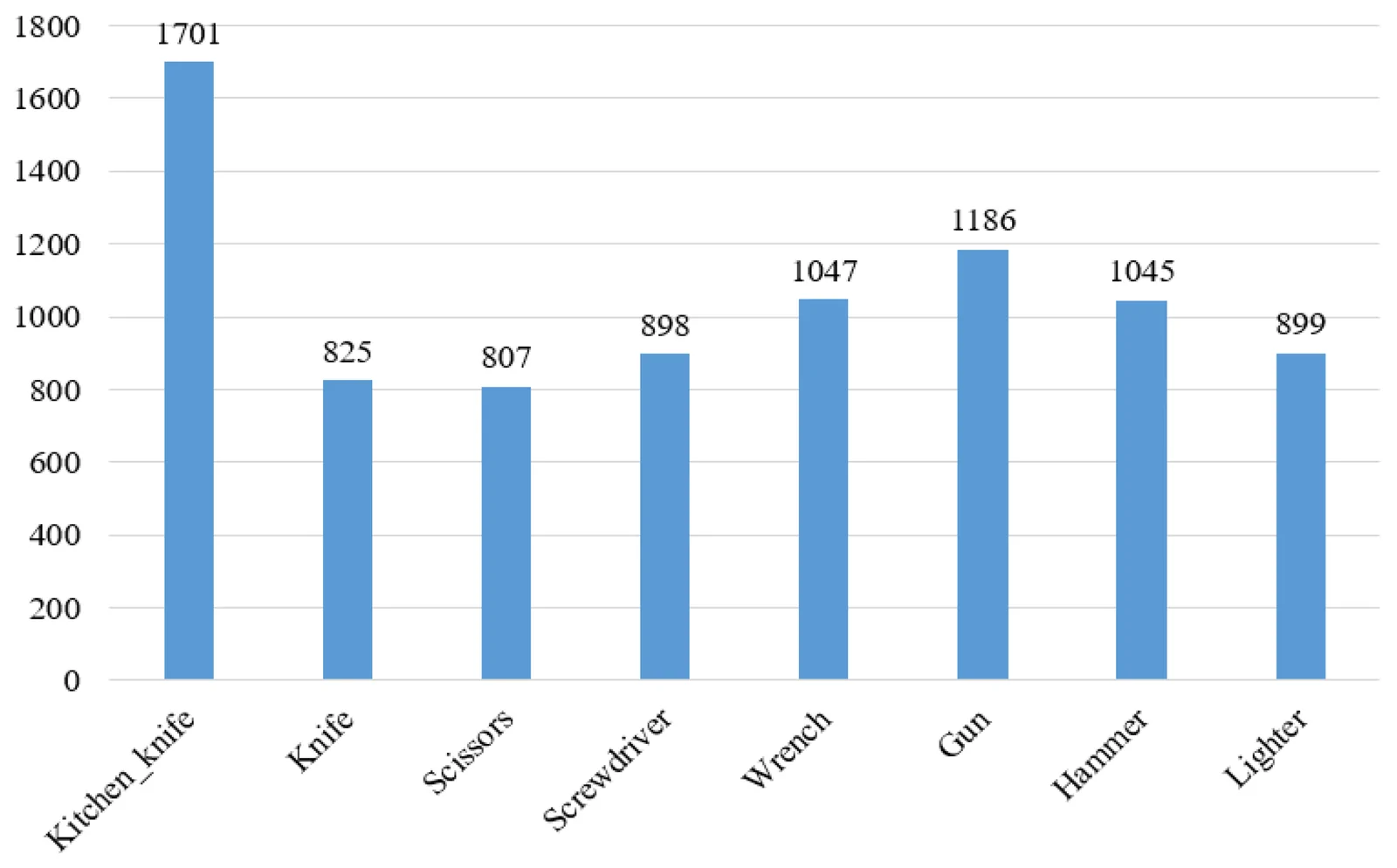
Instance distribution of each category in XPI8 Dataset.

The XPI8 Dataset includes eight prohibited-item categories, namely kitchen_knife, knife, scissors, screwdriver, wrench, gun, hammer, and lighter, with a total of 5015 annotated images. The instance counts for all categories are presented in Figure 12. No pseudo-color mapping or manually designed channel transformation was applied to the XPI8 images. To match the standard three-channel input of YOLO11n, the grayscale images were loaded using the default three-channel mode of the Ultralytics framework, resulting in three identical intensity channels.

### 3.2. Experimental Environment and Training Settings

The experiments were conducted on a Windows 11 workstation with an AMD Ryzen 7 8845H CPU, 16 GB RAM, and an NVIDIA GeForce RTX 4060 GPU. The models were implemented with PyTorch 2.0.0, CUDA 11.8, and Python 3.9. The maximum training epochs were set to 300 for SIXray and 200 for both OPIXray and XPI8, with an early-stopping patience of 20 epochs. The main training settings are summarized in Table 1.

During evaluation, all models were tested with an input resolution of 640 × 640 and a batch size of 32. The confidence threshold and Non-Maximum Suppression (NMS) IoU threshold were set to 0.001 and 0.5, respectively, with four data-loading workers. The same evaluation settings were used for all directly compared models within each dataset.

### 3.3. Evaluation Metrics

Model evaluation considers both detection performance and computational efficiency. *P* (Precision), *R* (Recall), *AP* (Average Precision), *mAP*@50 (mean Average Precision at IoU 0.5), and *mAP*@50:95 (mean Average Precision from IoU 0.5 to 0.95) are used as detection metrics, while Parameters, FLOPs (Floating Point Operations), and FPS (Frames Per Second) are adopted to characterize model complexity and inference efficiency.

P and R are adopted to measure detection quality, and their definitions are given in Equations (4) and (5):

(4) $$P=\frac{TP}{TP+FP}$$

(5) $$R=\frac{TP}{TP+FN}$$

where *TP*, *FP*, and *FN* represent true positives, false positives, and false negatives, respectively. For prohibited item screening, Recall is a critical metric because missed detections may directly affect inspection reliability.

AP jointly reflects Precision and Recall, and is defined as the area under the Precision–Recall curve, as shown in Equation (6):

(6) $$AP=\int_{0}^{1}P\left(R\right)\;dR$$

The mean *AP* over all categories is denoted as mAP, as expressed in Equation (7):

(7) $$mAP=\frac{1}{n}\sum_{i=1}^{n}{AP}_{i}$$

where *n* is the number of categories, and $AP_{i}$ denotes the *AP* of the i-th category.

For efficiency evaluation, Parameters and FLOPs are reported to measure model size and computational cost, whereas FPS is used to indicate inference speed in deployment. Since X-ray security inspection requires both accuracy and timely response, the trade-off among detection performance, model compactness, and processing speed is an important consideration in evaluating the proposed method.

## 4. Results and Analysis

### 4.1. Ablation Study

The ablation study was performed to clarify the individual and combined contributions of OSEM and BRSP to YOLO11-ORS on the SIXray dataset. Four configurations were evaluated: the original YOLO11n baseline, YOLO11n + OSEM, YOLO11n + BRSP, and YOLO11-ORS with both modules. To ensure a fair comparison, all variants used the same data partition, training protocol, and hyperparameter configuration. The quantitative results are listed in Table 2.

As shown in Table 2, OSEM and BRSP provide positive gains mainly in the *mAP* metrics, although their effects differ across specific evaluation measures. Compared with YOLO11n, OSEM increases *mAP*@50 from 92.9% to 93.4% and *mAP*@50:95 from 71.8% to 72.3%, corresponding to gains of 0.5 percentage points in both metrics. BRSP increases *mAP*@50 to 93.2% and *mAP*@50:95 to 72.5%, with gains of 0.3 and 0.7 percentage points, respectively. These results indicate that OSEM contributes to the representation of weak structural cues, whereas BRSP improves deep feature discrimination through semantic recalibration.

When OSEM and BRSP are used together, YOLO11-ORS increases Precision from 92.2% to 93.8%, *mAP*@50 from 92.9% to 94.1%, and *mAP*@50:95 from 71.8% to 72.7% compared with YOLO11n, corresponding to gains of 1.6, 1.2, and 0.9 percentage points, respectively. Recall changes only slightly from 89.3% to 89.1%. Meanwhile, the model remains compact with 2.68 M parameters and 6.4 GFLOPs. These results indicate that OSEM and BRSP provide complementary improvements in occlusion-oriented structural representation and background-response suppression.

### 4.2. Comparison of Different Attention Mechanisms

To examine whether the proposed occlusion-oriented attention design is more suitable for X-ray prohibited item detection, OSEM was compared with three representative attention modules, namely CBAM, SE, and ECA. Each module was inserted at the corresponding stage of the YOLOv11n backbone, while the data split, training schedule, and inference settings were kept unchanged. In this way, the performance differences can be mainly attributed to the attention design itself.

Table 3 shows that directly applying general-purpose attention mechanisms does not improve detection performance on SIXray. Compared with YOLOv11n, CBAM, SE, and ECA reduce *mAP*@50 from 92.9% to 90.9%, 91.3%, and 91.5%, respectively, and decrease *mAP*@50:95 from 71.8% to 66.7%, 66.7%, and 66.5%. In contrast, OSEM increases *mAP*@50 and *mAP*@50:95 to 93.4% and 72.3%, respectively, corresponding to gains of 0.5 percentage points over YOLO11n in both metrics.

In contrast, OSEM achieves better detection performance while keeping the model compact. More importantly, its improvement comes from a structure designed around the characteristics of occluded X-ray targets rather than from a general attention reweighting strategy. By considering local structural continuity and weak feature responses during feature transformation, OSEM is more suitable for handling partially occluded and overlapped prohibited items. Therefore, the comparison in Table 3 supports the use of an occlusion-oriented module instead of directly adopting conventional attention mechanisms.

### 4.3. Comparison with Representative Detection Methods

**Figure 13:**
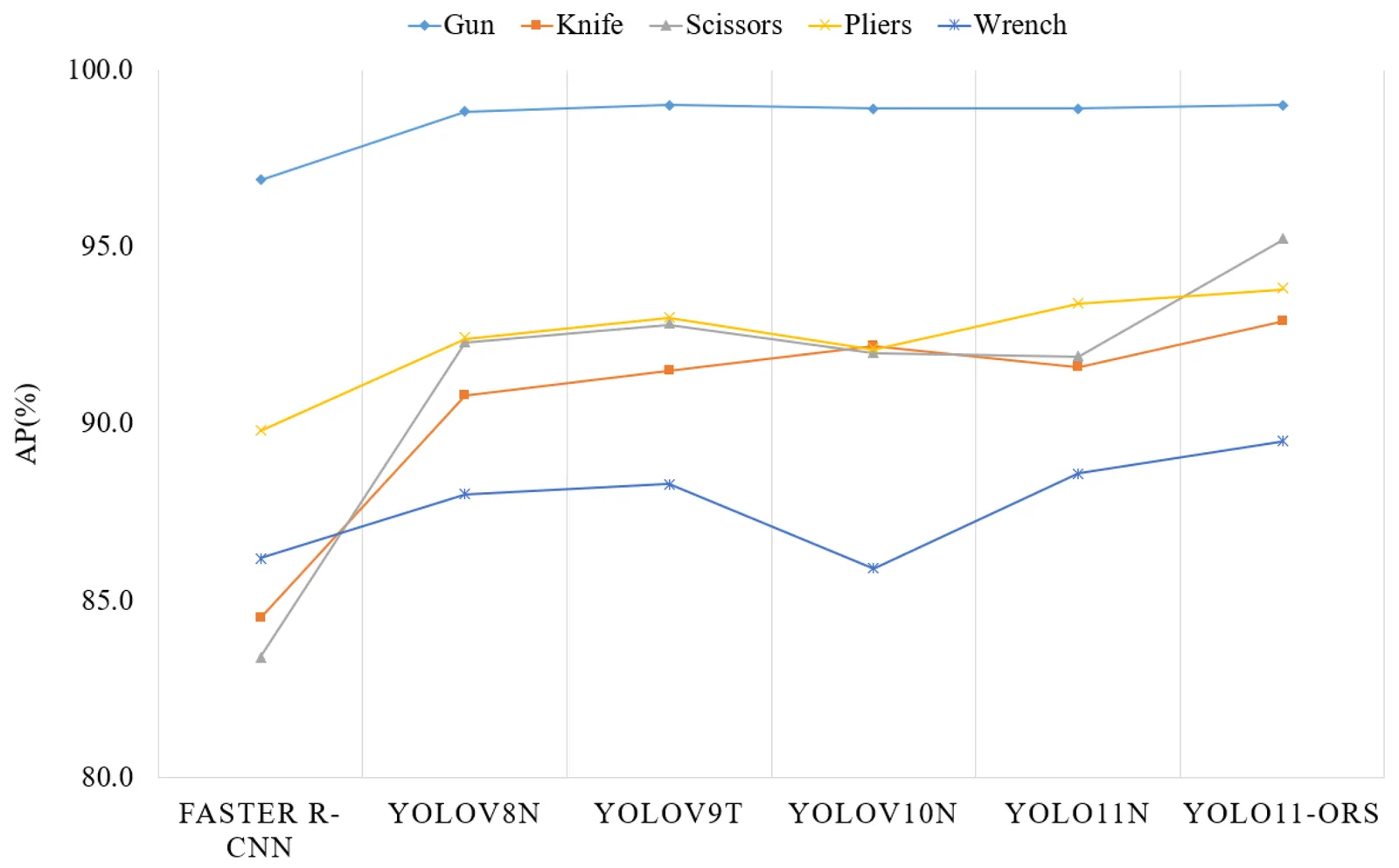
*AP* values of different prohibited item categories across detection models.

The proposed YOLO11-ORS was benchmarked against several representative detectors on the SIXray dataset, including Faster R-CNN, YOLOv8n, YOLOv9t, YOLOv10n, and YOLO11n. Table 4 summarizes the overall detection results and computational profiles, and Table 5 and Figure 13 provide the category-level *AP* comparison.

As shown in Table 4, compared with the YOLO11n baseline, YOLO11-ORS increases *mAP*@50 from 92.9% to 94.1% and *mAP*@50:95 from 71.8% to 72.7%, corresponding to improvements of 1.2 and 0.9 percentage points, respectively. Precision increases from 92.2% to 93.8%, while Recall changes slightly from 89.3% to 89.1%. The improvement in *mAP*@50:95 indicates that YOLO11-ORS not only enhances coarse object recognition but also improves localization stability under stricter IoU thresholds.

Compared with other lightweight YOLO variants, YOLO11-ORS achieves higher *mAP*@50 than YOLOv8n, YOLOv9t, and YOLOv10n by 1.6, 1.2, and 1.9 percentage points, respectively. For *mAP*@50:95, the corresponding improvements are 2.4, 0.2, and 1.3 percentage points, respectively. Although YOLOv9t has a smaller parameter scale and YOLOv10n provides the fastest inference speed, their detection accuracy is lower than that of YOLO11-ORS. Meanwhile, YOLO11-ORS maintains a compact profile with 2.68 M parameters, 6.4 GFLOPs, and 156 FPS, showing that the accuracy gain is achieved without a substantial increase in computational burden.

The category-level results in Table 5 further show that YOLO11-ORS improves *AP* across all five prohibited-item categories compared with YOLO11n. The *AP* gains for Gun, Knife, Scissors, Pliers, and Wrench are 0.1, 1.3, 3.3, 0.4, and 0.9 percentage points, respectively. Among them, the improvement is most evident for Scissors, whose *AP* increases from 91.9% to 95.2%. This category commonly exhibits thin and fragmented structural cues in X-ray images and can be strongly affected by object overlap and surrounding interference. The larger improvement for Scissors, together with the positive gains observed in the other categories, indicates that the proposed feature-enhancement and background-recalibration mechanisms can improve the representation of visually ambiguous prohibited items.

Overall, the comparison demonstrates that YOLO11-ORS improves detection accuracy on SIXray while keeping the model suitable for real-time inference. The results also indicate that the proposed occlusion-oriented and background-suppression designs provide a more favorable accuracy–efficiency trade-off than directly using the YOLO11n baseline or other representative lightweight detectors.

### 4.4. Performance on Difficult Test Subsets

To assess the robustness of YOLO11-ORS in more challenging X-ray scenarios, experiments were further performed on the selected difficult subsets of SIXray. Four model variants were evaluated, including the original YOLO11n baseline, YOLO11n equipped with OSEM, YOLO11n equipped with BRSP, and the final YOLO11-ORS. All variants followed the same evaluation protocol, and the quantitative results are reported in Table 6.

To assess the robustness of YOLO11-ORS under challenging X-ray conditions, the four model variants were further evaluated on the difficult subsets of SIXray, as reported in Table 6. On the cluttered-background subset, YOLO11-ORS improves *mAP*@50 by 1.4 percentage points over YOLO11n, while the gain in *mAP*@50:95 is relatively limited. BRSP alone increases Recall by 1.6 percentage points, indicating that semantic recalibration helps distinguish target-related responses from redundant background structures. On the overlap subset, YOLO11-ORS provides more evident gains, with Precision, *mAP*@50, and *mAP*@50:95 increasing by 2.8, 1.7, and 0.9 percentage points, respectively, while Recall remains nearly unchanged. This suggests that the proposed modules mainly improve detection reliability and localization quality when target features are entangled with overlapping objects.

For the small-object subset, the improvement is comparatively moderate: YOLO11-ORS increases *mAP*@50 and *mAP*@50:95 by 1.0 and 0.4 percentage points, respectively, while Recall decreases slightly. This indicates that the proposed design can improve the quality of small-target predictions but is not specifically optimized for recovering missed small objects. In contrast, on the merged difficult subset, YOLO11-ORS achieves simultaneous gains in Precision, Recall, *mAP*@50, and *mAP*@50:95 of 1.2, 0.8, 1.7, and 0.8 percentage points, respectively. The more consistent improvement under mixed challenging conditions reflects the complementary roles of OSEM in preserving weak and incomplete target structures and BRSP in suppressing redundant interfering responses.

**Figure 14:**
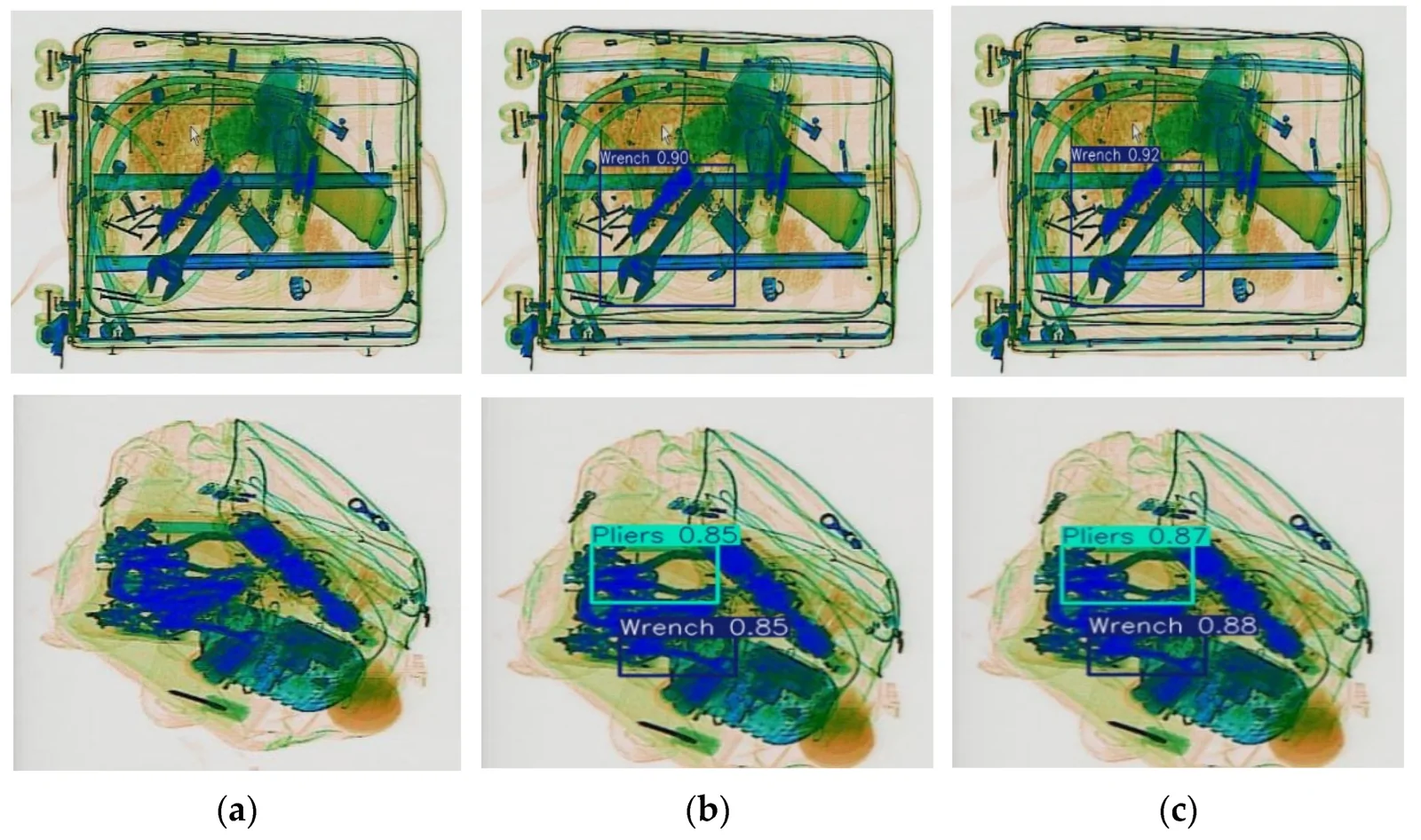
Detection results of YOLO11n and YOLO11-ORS on difficult samples. (**a**) Original image; (**b**) YOLO11n; (**c**) YOLO11-ORS.

The qualitative comparisons in Figure 14 are consistent with these quantitative trends. Compared with YOLO11n, YOLO11-ORS better preserves target-related cues and reduces interference from surrounding structures in overlapping and cluttered scenes, further supporting the effectiveness of the coordinated OSEM and BRSP design.

**Figure 15:**
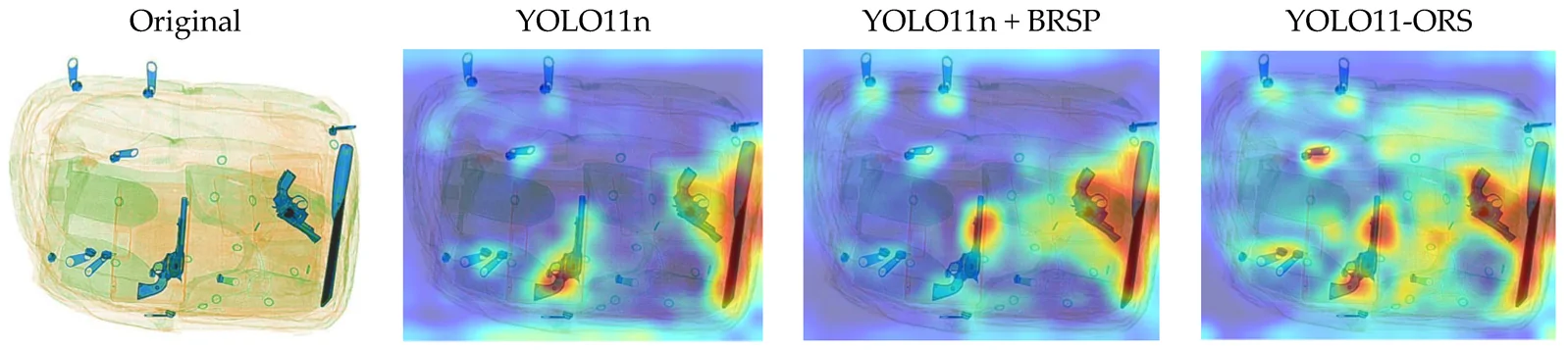
Visualization of deep feature responses for background redundancy suppression on a representative cluttered-background sample from SIXray. From left to right: original X-ray image, YOLO11n, YOLO11n + BRSP, and YOLO11-ORS.

To further investigate the effect of BRSP on feature representation under cluttered backgrounds, Figure 15 visualizes the deep feature responses of YOLO11n, YOLO11n + BRSP, and YOLO11-ORS on a representative SIXray sample. Compared with the baseline, introducing BRSP noticeably redistributes the spatial feature responses, with high-response regions becoming more concentrated around prohibited items and other discriminative structures. In particular, the responses associated with the gun-shaped targets become more prominent, whereas several diffuse responses over less informative baggage regions are weakened or reorganized. YOLO11-ORS maintains this target-oriented response pattern while highlighting multiple prohibited-item regions. These qualitative observations are consistent with the intended role of BRSP in recalibrating aggregated semantic features and reducing interference from cluttered background structures.

### 4.5. Evaluation on the OPIXray Dataset

To further examine the effectiveness of the proposed method on an independent public X-ray benchmark and its robustness to target occlusion, YOLO11n, YOLO11n + OSEM, YOLO11n + BRSP, and YOLO11-ORS were evaluated on OPIXray. The overall results are reported in Table 7.

Compared with YOLO11n, introducing OSEM increases *mAP*@50 and *mAP*@50:95 by 0.7 and 0.4 percentage points, respectively, while BRSP produces corresponding gains of 0.9 and 0.4 percentage points. When the two modules are combined, YOLO11-ORS improves Precision and Recall by 1.2 and 0.9 percentage points and increases *mAP*@50 and *mAP*@50:95 by 1.2 and 0.8 percentage points, respectively. These results indicate that the improvements obtained on SIXray can also be observed on OPIXray, providing additional evidence that the proposed modifications are not specific to a single X-ray dataset.

To further investigate the influence of target occlusion, the models were evaluated separately on OL1, OL2, and OL3. Since the purpose of this comparison is to analyze detection and localization performance under different occlusion levels, only *mAP*@50 and *mAP*@50:95 are reported in Table 8.

Compared with YOLO11n, OSEM improves *mAP*@50:95 across all three occlusion levels, with a more evident improvement for OL2. This observation is consistent with its intended role in preserving weak and incomplete structural information when targets are partially occluded. BRSP exhibits a less consistent gain across different occlusion levels. This is mainly related to its design objective: BRSP recalibrates multi-scale semantic features to reduce redundant responses and improve target–interference separability, but it does not explicitly restore structural cues lost because of occlusion. As the occlusion becomes more severe, incomplete and fragmented target representations become the dominant difficulty, which limits the benefit of background-oriented semantic recalibration alone. Nevertheless, the improvement in *mAP*@50:95 on OL2 and OL3 indicates that BRSP can still contribute to localization stability under stricter IoU criteria.

For YOLO11-ORS, the change relative to YOLO11n becomes particularly evident under the stricter localization metric. Its *mAP*@50:95 increases by 0.7, 0.7, and 1.5 percentage points on OL1, OL2, and OL3, respectively. In particular, the larger improvement for OL3 indicates that the proposed combination maintains more stable localization performance when the visible target structure is severely degraded. Taken together with the SIXray difficult-subset results, the OPIXray experiments provide complementary evidence for the effectiveness of the proposed occlusion-oriented feature representation under challenging X-ray imaging conditions.

**Figure 16:**
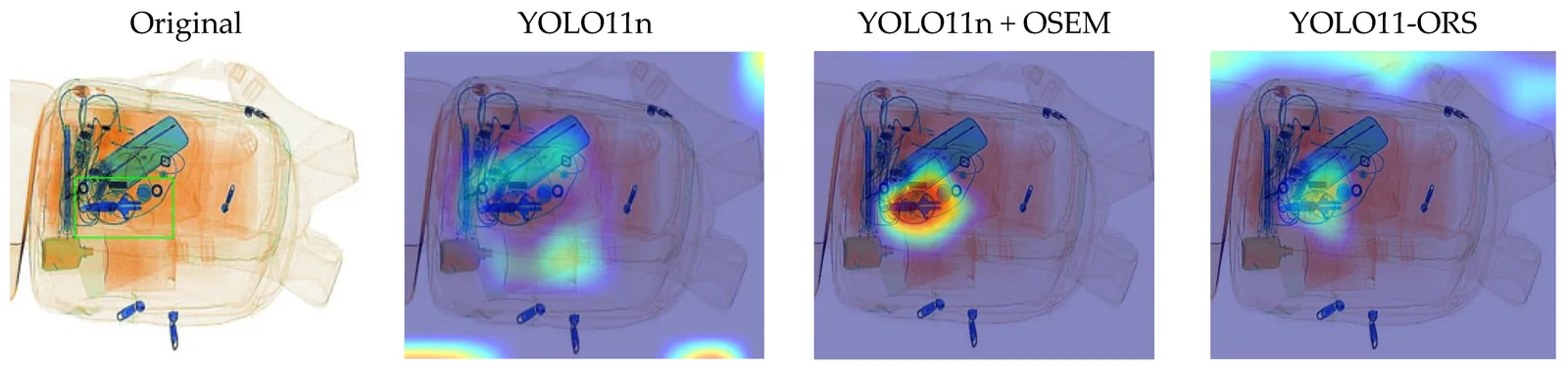
Class-specific Grad-CAM visualization for an occluded prohibited item on the OPIXray dataset. From left to right: original X-ray image, YOLO11n, YOLO11n + OSEM, and YOLO11-ORS. The green bounding box indicates the ground-truth target used to determine the visualization category.

To further examine how OSEM affects the discriminative evidence used for recognizing occluded prohibited items, Figure 16 presents class-specific Grad-CAM visualizations for a representative OPIXray sample. Compared with YOLO11n, the model equipped with OSEM exhibits a more concentrated class-discriminative response within and around the ground-truth target region, while the baseline response is more spatially dispersed. YOLO11-ORS retains a clear response over the target-related region. These qualitative observations suggest that OSEM encourages the detector to rely more strongly on discriminative structural cues associated with the occluded prohibited-item class, which is consistent with the improved localization performance under different occlusion levels reported in Table 8.

Failure-oriented analysis further reveals several remaining limitations of the proposed model. On the SIXray small-object subset, although YOLO11-ORS improves *mAP*@50 from 90.9% to 91.9% and *mAP*@50:95 from 64.9% to 65.3%, Recall decreases slightly from 86.6% to 85.9%. This indicates that the proposed feature-enhancement strategy can improve the quality of detected small-object predictions but remains limited in recovering extremely small or weakly visible targets. A similar limitation can be observed under severe occlusion on OPIXray. On OL3, YOLO11-ORS improves *mAP*@50:95 from 40.2% to 41.7%, whereas *mAP*@50 changes slightly from 88.7% to 88.5%. This suggests that the proposed method improves localization robustness for successfully detected targets under severe occlusion, but cannot fully compensate for the substantial loss of visible structural information. Therefore, extremely small targets and severely occluded objects remain challenging cases for the current framework.

### 4.6. Validation on the Self-Collected Dataset

To examine the applicability of YOLO11-ORS under a different X-ray imaging modality, an additional validation experiment was carried out on the XPI8 Dataset. YOLO11n was used as the baseline, and both YOLO11n and YOLO11-ORS were trained and evaluated using the same optimization and model-selection settings. The category-level *AP* results are presented in Table 9.

As shown in Table 9, both YOLO11n and YOLO11-ORS achieve very high *mAP*@50 values on the XPI8 Dataset. The baseline YOLO11n obtains 99.3% *mAP*@50, while YOLO11-ORS reaches 99.4% *mAP*@50. The proposed model shows slight improvements in most categories, including kitchen_knife, knife, scissors, screwdriver, wrench, hammer, and lighter. Although the *AP* of Gun decreases slightly from 99.2% to 98.9%, the overall *mAP*@50 remains marginally higher than that of the baseline.

Since the XPI8 Dataset contains grayscale images with simpler scene structures and lower background complexity than SIXray, it is used here as supplementary evidence for cross-imaging validation rather than as the main basis for claiming model superiority. The experimental results show that introducing OSEM and BRSP does not reduce the overall detection capability of YOLO11n when the imaging modality changes. This suggests that YOLO11-ORS can maintain consistent performance across dual-energy pseudo-color X-ray images and single-energy grayscale X-ray images, although the two datasets differ in background complexity and visual appearance.

## 5. Conclusions

This study proposed YOLO11-ORS, an improved YOLOv11-based network for prohibited item detection in X-ray security images. The model addresses the difficulties caused by object overlap, partial occlusion, weak target boundaries, and cluttered baggage backgrounds by enhancing semantic feature discrimination and preserving weak structural cues of occluded targets. Experimental results demonstrate that the proposed method improves detection accuracy and robustness in complex X-ray inspection scenarios while maintaining practical inference efficiency. Additional evaluation on OPIXray shows that the improvement in *mAP*@50:95 is maintained across different occlusion levels, with the relative gain increasing to 1.5 percentage points under severe occlusion (OL3). Nevertheless, several limitations remain. The difficult-subset analysis shows that the improvement for extremely small or weakly visible targets is relatively limited, and Recall decreases slightly on the SIXray small-object subset. Under severe occlusion, although YOLO11-ORS improves *mAP*@50:95, the *mAP*@50 gain is not maintained, indicating that the current feature-enhancement strategy cannot fully compensate for substantial loss of visible target information. The XPI8 results further provide supplementary evidence of model applicability under a different X-ray imaging modality. Nevertheless, the gains remain limited in some challenging cases, and the relatively simple scene composition of XPI8 leads to a noticeable ceiling effect, limiting its ability to distinguish between high-performing detectors.

Future work will therefore focus on three directions. First, more diverse and challenging X-ray datasets will be incorporated, together with finer-grained occlusion and small-target annotations, to improve evaluation under extreme visibility degradation. Second, feature representations specifically designed for extremely small and severely occluded targets will be investigated. Third, future work will also consider more recent detection architectures as base models, allowing the proposed feature-optimization strategies to be further evaluated and extended along with advances in object detection frameworks.

## Figures and Tables

**Table 1:** Main training configuration.

Parameter	Setting	Parameter	Setting
Maximum epochs	SIXray: 300; OPIXray: 200; XPI8: 200.	Early-stopping patience	20
Image size	640 × 640	Batch size	32
Workers	4	Optimizer	SGD
*lr0*	0.01	*lrf*	0.01
Momentum	0.937	Weight decay	0.0005
Warm-up epochs	3	AMP	Enabled
Data augmentation	Default Ultralytics augmentation settings		

**Table 2:** Ablation results of module configurations on the SIXray dataset.

OSEM	BRSP	*P* (%)	*R* (%)	*mAP*@50 (%)	*mAP*@50:95 (%)	Parameters (M)	FLOPs (G)	FPS
		92.2	89.3	92.9	71.8	2.73	6.6	145
√		92.1	89.1	93.4	72.3	2.54	6.3	163
	√	93.4	89.1	93.2	72.5	2.72	6.4	164
√	√	93.8	89.1	94.1	72.7	2.68	6.4	156

**Table 3:** Ablation of different attention designs in the YOLOv11n backbone.

Model	*P* (%)	*R* (%)	*mAP*@50 (%)	*mAP*@50:95 (%)	Parameters (M)	FLOPs (G)	FPS
YOLO11n	92.2	89.3	92.9	71.8	2.73	6.6	145
+CBAM	91.3	85.9	90.9	66.7	3.39	10.8	110
+SE	91.0	85.9	91.3	66.7	3.44	9.8	135
+ECA	91.4	84.2	91.5	66.5	3.43	10.6	143
+OSEM	92.1	89.1	93.4	72.3	2.54	6.3	163

**Table 4:** Overall evaluation of representative detectors on the SIXray dataset.

Model	*P* (%)	*R* (%)	*mAP*@50 (%)	*mAP*@50:95 (%)	Parameters (M)	FLOPs (G)	FPS
Faster R-CNN	53.2	58.7	88.2	60.0	41.37	216.5	12
YOLOv8n	93.2	87.8	92.5	70.3	3.01	8.1	149
YOLOv9t	93.7	88.5	92.9	72.5	1.97	7.6	179
YOLOv10n	91.6	87.3	92.2	71.4	2.27	6.5	244
YOLO11n	92.2	89.3	92.9	71.8	2.73	6.6	145
YOLO11-ORS	93.8	89.1	94.1	72.7	2.68	6.4	156

**Table 5:** Category-level *AP* results of different detectors on the SIXray dataset.

Model	Gun	Knife	Scissors	Pliers	Wrench	*mAP*@50 (%)
Faster R-CNN	96.9	84.5	83.4	89.8	86.2	88.2
YOLOv8n	98.8	90.8	92.3	92.4	88.0	92.5
YOLOv9t	99.0	91.5	92.8	93.0	88.3	92.9
YOLOv10n	98.9	92.2	92.0	92.1	85.9	92.2
YOLO11n	98.9	91.6	91.9	93.4	88.6	92.9
YOLO11-ORS	99.0	92.9	95.2	93.8	89.5	94.1

**Table 6:** Performance comparison on selected difficult test subsets of SIXray.

Subset	Model	*P* (%)	*R* (%)	*mAP*@50 (%)	*mAP*@50:95 (%)
	YOLO11n	94.9	92.0	95.1	77.0
Cluttered background	YOLO11n + OSEM	95.9	93.4	96.2	77.9
	YOLO11n + BRSP	93.9	93.6	95.4	77.5
	YOLO11-ORS	95.0	92.9	96.5	77.1
	YOLO11n	90.6	87.5	91.3	69.1
Overlap	YOLO11n + OSEM	91.3	88.6	93.0	69.4
	YOLO11n + BRSP	91.9	87.2	92.2	70.0
	YOLO11-ORS	93.4	87.4	93.0	70.0
	YOLO11n	91.4	86.6	90.9	64.9
Small object	YOLO11n + OSEM	90.3	87.2	91.7	65.2
	YOLO11n + BRSP	92.6	85.6	90.9	64.8
	YOLO11-ORS	92.7	85.9	91.9	65.3
	YOLO11n	92.3	87.2	91.4	69.4
All difficult	YOLO11n + OSEM	91.1	88.4	92.6	69.9
	YOLO11n + BRSP	93.2	87.6	92.2	69.8
	YOLO11-ORS	93.5	88.0	93.1	70.2

**Table 7:** Overall detection performance on the OPIXray test set.

Model	*P* (%)	*R* (%)	*mAP*@50 (%)	*mAP*@50:95 (%)
YOLO11n	89.5	86.2	88.9	42.1
YOLO11n + OSEM	89.0	85.5	89.6	42.5
YOLO11n + BRSP	91.2	85.3	89.8	42.5
YOLO11-ORS	90.7	87.1	90.1	42.9

**Table 8:** Detection performance under different occlusion levels on OPIXray.

Model	OL1 *mAP*@50 (%)	OL1 *mAP*@50:95 (%)	OL2 *mAP*@50 (%)	OL2 *mAP*@50:95 (%)	OL3 *mAP*@50 (%)	OL3 *mAP*@50:95 (%)
YOLO11n	89.5	43.6	88.3	41.3	88.7	40.2
YOLO11n + OSEM	89.8	43.8	89.5	42.0	88.8	40.6
YOLO11n + BRSP	91.5	43.6	88.5	41.9	87.5	41.6
YOLO11-ORS	92.0	44.3	88.1	42.0	88.5	41.7

**Table 9:** Comparative results on XPI8 Dataset.

Model	Kitchen_Knife	Knife	Scissors	Screwdriver	Wrench	Gun	Hammer	Lighter	*mAP*@50 (%)
YOLO11n	99.4	99.3	99.1	99.3	99.3	99.2	99.4	99.5	99.3
YOLO11-ORS	99.5	99.5	99.4	99.5	99.5	98.9	99.5	99.5	99.4

## Data Availability

The SIXray dataset used in this study is openly available in the SIXray repository at https://github.com/MeioJane/SIXray (accessed on 9 August 2026). The OPIXray dataset is also publicly available at https://github.com/OPIXray-author/OPIXray (accessed on 10 August 2026). The complete SIXray difficult test subsets constructed in this study, including the small-object, overlap, cluttered-background, and merged overall difficult subsets, together with the corresponding image-index files and dataset notes, are publicly available at https://github.com/Warren318/Difficult_Test_Subsets_Dataset/releases/tag/v1.0 (accessed on 10 June 2026). The XPI8 Dataset analyzed in this study is not publicly available due to privacy and intellectual property restrictions, but is available upon reasonable request from the corresponding author.

## Abbreviations

The following abbreviations are used in this manuscript:

*AP*	Average Precision
BRSP	Background Redundancy Suppression Pyramid
C2PSA	Cross Stage Partial with Spatial Attention
CBAM	Convolutional Block Attention Module
CHR	Class-balanced Hierarchical Refinement
CNN	Convolutional Neural Network
FFN	Feed-forward Network
FLOPs	Floating Point Operations
FPS	Frames Per Second
GCE	Grouped Convolutional Block Attention Enhancement
GELU	Gaussian Error Linear Unit
GFLOPs	Giga Floating Point Operations
IoU	Intersection over Union
*mAP*	mean Average Precision
OSEM	Occluded Structure Enhancement Module
ORS	Occlusion Representation and Background Redundancy Suppression
PSABlock	Spatial Attention Block
R-CNN	Region-based Convolutional Neural Network
SE	Squeeze-and-Excitation
SEAM	Separation and Enhancement Attention Module
SGD	Stochastic Gradient Descent
SiLU	Sigmoid Linear Unit
SPPF	Spatial Pyramid Pooling Fast
SSD	Single Shot MultiBox Detector
TP	True Positive
FP	False Positive
FN	False Negative
XPI8	X-ray Prohibited-item Dataset with 8 categories
YOLO	You Only Look Once
